# Supplementary material for: Varying the position of phospholipid acyl chain unsaturation modulates hopanoid and sterol ordering
Source: Biophys J. 2024 Jun 6;123(13):1896–902. doi: 10.1016/j.bpj.2024.06.002 (PMC11267422; doi:10.1016/j.bpj.2024.06.002)

Biophysical Journal, Volume *123*

## Supplemental information

# Varying the position of phospholipid acyl chain unsaturation modulates hopanoid and sterol ordering

**Ha Ngoc Anh Nguyen, Liam Sharp, Edward Lyman, and James P. Saenz**

# Supplemental Data

**Fig S1. Final snapshots of snapshots from Dpop simulations.** Left panel: 50:50 mixture of Dpop and Δ9-DOPC. Right panel: 50:50 mixture of Dpop and Δ11-DOPC. Phospholipids are rendered in gray with blue spheres marking the phosphates, and Dpop is rendered in cyan with red spheres marking the hydroxyl.

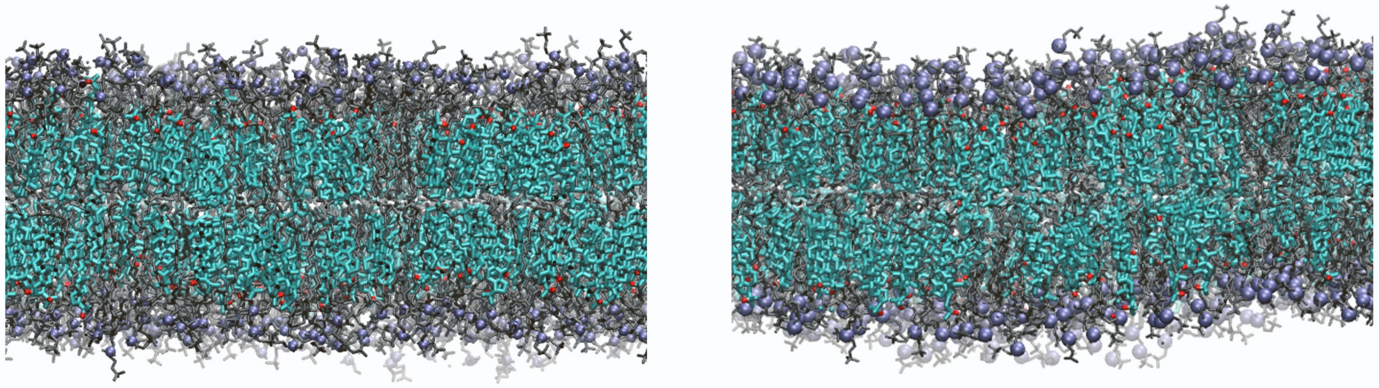

**Fig S2. Raw data of 3 monolayers isotherm replicates.**

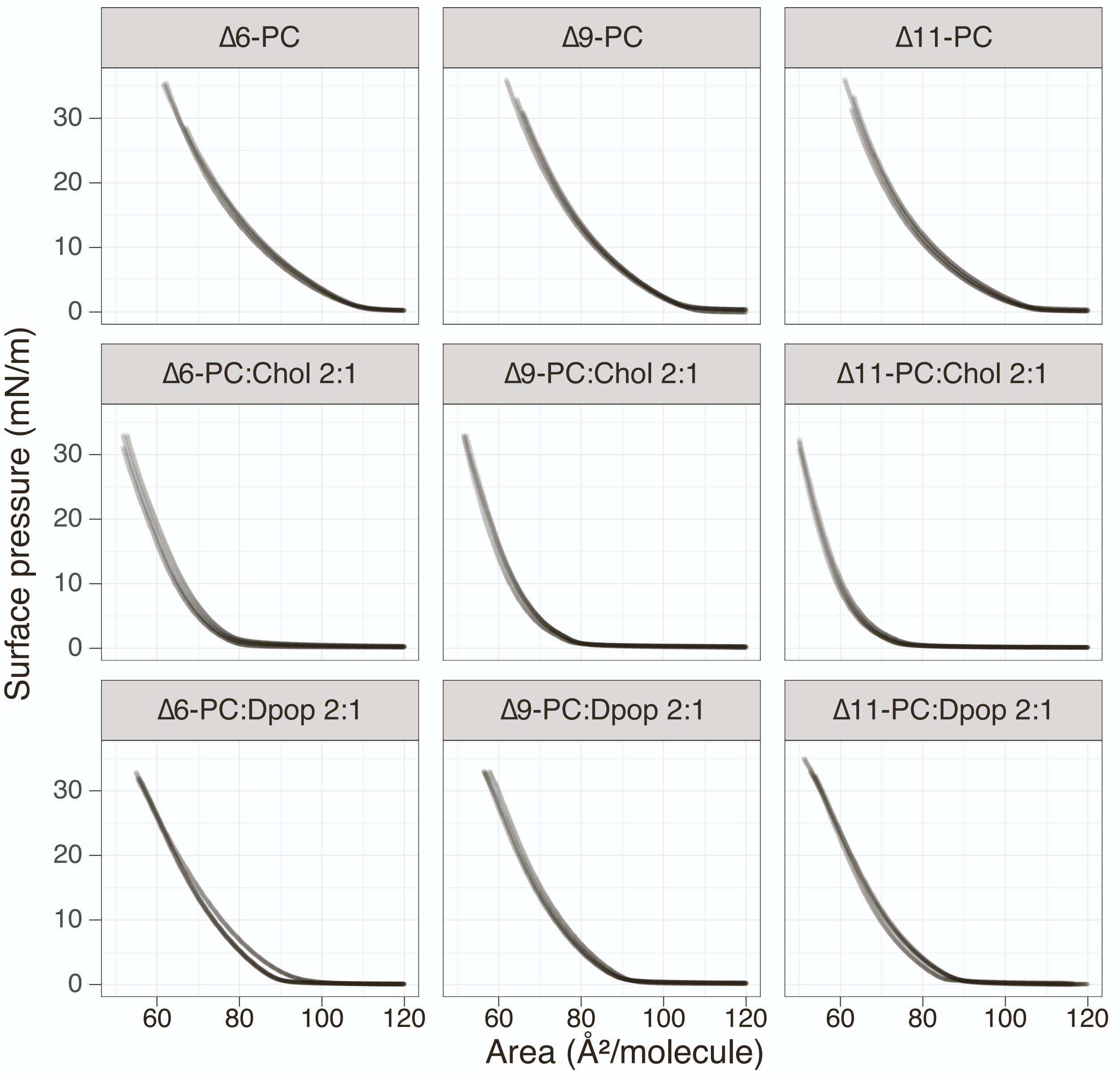

The isotherms were fitted to regression for statistical analysis with the following formula:

$$\text{Surface pressure} = \text{coef1} \times \frac{1}{\text{Area}^2} + \text{coef2}$$

**Table S1. Coefficients calculated for each isotherm**

| Sample | Pure | | 1:2 Chol:PC | | 1:2 Dpop:PC | |
|---|---|---|---|---|---|---|
| | coef1 | coef2 | coef1 | coef2 | coef1 | coef2 |
| Δ6-PC | 8.93E-05 | 4.87E-06 | 1.77E-04 | 6.25E-06 | 1.24E-04 | 5.91E-06 |
| Δ6-PC | 8.32E-05 | 5.07E-06 | 1.73E-04 | 6.02E-06 | 1.22E-04 | 6.10E-06 |
| Δ6-PC | 8.33E-05 | 4.89E-06 | 1.66E-04 | 5.85E-06 | 1.09E-04 | 6.49E-06 |
| Δ9-PC | 9.65E-05 | 4.64E-06 | 1.95E-04 | 5.67E-06 | 1.29E-04 | 5.47E-06 |
| Δ9-PC | 9.51E-05 | 4.50E-06 | 1.84E-04 | 5.84E-06 | 1.27E-04 | 5.43E-06 |
| Δ9-PC | 9.44E-05 | 4.43E-06 | 1.85E-04 | 5.58E-06 | 1.25E-04 | 5.16E-06 |
| Δ11-PC | 1.01E-04 | 4.75E-06 | 2.25E-04 | 5.75E-06 | 1.27E-04 | 6.62E-06 |
| Δ11-PC | 1.02E-04 | 4.99E-06 | 2.19E-04 | 5.77E-06 | 1.34E-04 | 6.24E-06 |
| Δ11-PC | 9.70E-05 | 4.77E-06 | 2.12E-04 | 5.88E-06 | 1.40E-04 | 6.37E-06 |

**Fig S3**. Structures of cholesterol (left) and diplopterol (right), with atoms used to define molecular orientation labeled.

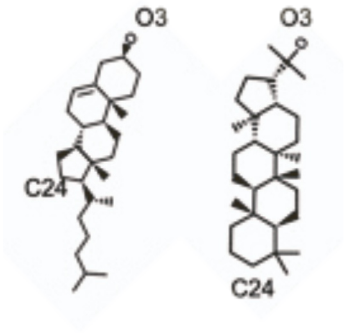


**Fig S4**. Example growth curve through OD562nm read-out. The slope of the curve from 0.75 to 0.4 was used to calculate the growth rate (a.u.)

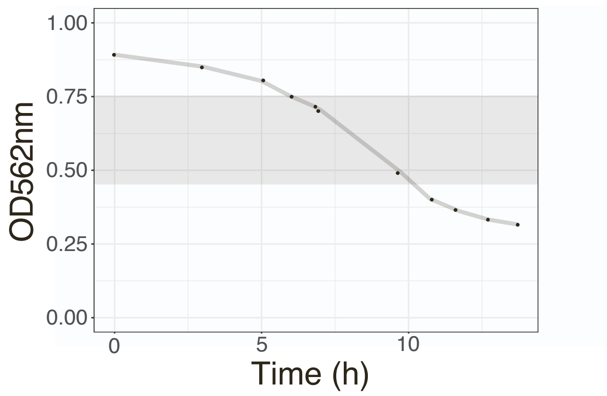

Supplement: Document S1. Table S1 and Figures S1–S4 [file mmc1.pdf]
