# Supplementary material for: Varying the position of phospholipid acyl chain unsaturation modulates hopanoid and sterol ordering
Source: Biophys J. 2024 Jun 6;123(13):1896–902. doi: 10.1016/j.bpj.2024.06.002 (PMC11267422; doi:10.1016/j.bpj.2024.06.002)

Biophysical Journal
Article

Biophysical Society

Varying the position of phospholipid acyl chain unsaturation modulates hopanoid and sterol ordering

Ha Ngoc Anh Nguyen,[1] Liam Sharp,[2,3] Edward Lyman,[2,4] and James P. Saenz[1,5,*]
[1]Technische Universität Dresden, B CUBE Center for Molecular Bioengineering, Dresden, Germany; [2]Department of Physics and Astronomy, University of Delaware, Newark, Delaware; [3]College of Arts and Sciences, Fairfield University, Fairfield, Connecticut; [4]Department of Chemistry and Biochemistry, University of Delaware, Newark, Delaware; and [5]Medical Faculty, Technische Universität Dresden, Dresden, Germany

ABSTRACT The cell membrane must balance mechanical stability with fluidity to function as both a barrier and an organizational platform. Key to this balance is the ordering of hydrocarbon chains and the packing of lipids. Many eukaryotes synthesize sterols, which are uniquely capable of modulating the lipid order to decouple membrane stability from fluidity. Ancient sterol analogs known as hopanoids are found in many bacteria and proposed as ancestral ordering lipids. The juxtaposition of sterols and hopanoids in extant organisms prompts us to ask why both pathways persist, especially in light of their convergent ability to order lipids. In this work, simulations, monolayer experiments, and cellular assays show that hopanoids and sterols order unsaturated phospholipids differently based on the position of double bonds in the phospholipid acyl chain. We find that cholesterol and diplopterol's methyl group distributions lead to distinct effects on unsaturated lipids. In *Mesoplasma florum*, diplopterol's constrained ordering capacity reduces membrane resistance to osmotic stress, unlike cholesterol. These findings suggest that cholesterol's broader lipid-ordering ability may have facilitated the exploration of a more diverse lipidomic landscape in eukaryotic membranes.

SIGNIFICANCE This study explores how the diversity of isoprenoid structures, specifically sterols and hopanoids, has influenced membrane composition across domains of life. By comparing the divergent interaction of these compounds with phospholipids, we show that cholesterol, unlike its ancient counterpart diplopterol, effectively orders a broader range of lipids with varying double bond positions. The molecular versatility of cholesterol may have been key to the development of complex eukaryotic lipidomes, facilitating diverse cellular functions. Our findings not only highlight the evolutionary significance of membrane isoprenoids but also suggest a mechanism by which hopanoid-containing organisms may modulate lipid ordering by tuning the double bond position. This study bridges membrane evolution and biophysics, offering insights into the physical basis of lipid diversity.

INTRODUCTION

To support life, cell membranes must balance mechanical stability (sufficient to perform robustly as a barrier) against fluidity and deformability (sufficient to support its role as an organizational platform for bioactivity). However, for bilayer-forming mixtures of lipids, stability is often gained at the expense of fluidity. Different solutions to this dilemma have evolved on different branches of the tree of life. For example, thermophilic archaea synthesize double-headed bolalipids that maintain membrane integrity even at extreme temperatures (1). In many eukaryotes, sterols solve this problem: their twin faces simultaneously order hydrocarbon chains while promoting lateral diffusivity (2). By decoupling the local motion of acyl chains and lipid translational freedom of motion, sterols allow cells to build membranes that are mechanically stable enough to withstand environmental perturbations but fluid enough to support diffusion-dependent biochemistry.

Most noneukaryotic organisms cannot synthesize sterols and must rely on other mechanisms to modulate their membrane properties. Some bacteria utilize a family of compounds called hopanoids (3). Hopanoids are tri-terpenoids, found in the sedimentary record as early as 1.64 billion years ago (4). Since both families are synthesized from squalene, with homologous enzymes (squalene-hopene

Submitted February 23, 2024, and accepted for publication June 4, 2024.
*Correspondence: james.saenz@tu-dresden.de
Editor: Ilya Levental.
https://doi.org/10.1016/j.bpj.2024.06.002
© 2024 Biophysical Society.
This is an open access article under the CC BY license (http://creativecommons.org/licenses/by/4.0/).

cyclase and oxidosqualene cyclase) (5), they share certain chemical similarities. Like sterols, hopanoids also reside within the membrane and modulate membrane robustness, fluidity, and resistance against abiotic stresses (3,6). For these reasons, hopanoids are considered both bacterial and ancient sterol analogs. But why does life need two analogous classes of ordering lipids?

Despite their similarities, hopanoids and sterols possess distinct properties. Both diplopterol (Dpop), a common hopanoid in bacteria, and cholesterol (Chol), a mammalian sterol, interact favorably with and condense saturated lipids, a diagnostic feature of lipid ordering (7). However, the condensing effect of Dpop is impaired when an acyl chain unsaturation is introduced and in a manner that depends on the location of the double bond. In this work, we examined how a double bond position influences sterol and hopanoid ordering. We find that methyl group distribution on hopanoid backbones restricts its ordering ability, resulting in pronounced effects on cellular robustness in the model organism *Mesoplasma florum*. Conversely, Chol ordering is comparatively insensitive to the double bond position, allowing eukaryotic membranes more chemical flexibility in regulating their lipidomes.

MATERIALS AND METHODS

Materials

Δ6-, Δ9-, and Δ11-PC and egg sphingomyelin were purchased from Avanti Polar Lipids (Alabaster, AL, USA). Chol and palmitic acid were purchased from Sigma (St. Louis, MO, USA) and Dpop from Chiron (Tuttlingen, Germany). Stock concentrations of lipids were measured by phosphate assay. Chol and Dpop were weighed out on a precision scale and solubilized in a known volume of chloroform.

Methods

Monolayer

Chloroform solutions of pure lipids and mixtures were prepared at 0.2 mg/mL lipid concentrations. Monolayers were prepared by injecting 15–30 μL of lipid solution onto an aqueous subphase maintained at 20°C by a built-in temperature-controlled circulating water bath. The subphase was comprised of 10 mM HEPES, 150 mM NaCl (pH 7). Isotherms were recorded using a 70 cm^2 Teflon Langmuir trough fitted with a motorized compression barrier equipped with a pressure sensor (Kibron DeltaPi).

The mean molecular areas (MMAs) for each mixture was estimated from the averages of isotherms from three monolayers that were prepared independently. Data were rounded down to the nearest neighbor for the condensation effect and free energy calculations. All isotherms were fitted to a regression, and statistical significance was tested using MANOVA (multivariate analysis of variance) with the two coefficients.

The condensation effect was calculated as follows:

$$c = 100 - \frac{A_0}{X_1A_1+X_2A_2}\,(\%) \qquad \text{(Equation 1)}$$

where c = % condensation, A_o = the MMA of the lipid mixture, X_1 and X_2 = the mole fractions of lipids 1 and 2 in the mix, and A_1 and A_2 = the MMAs of lipids 1 and 2 at surface pressures 30 mN/m. Error bars were produced based on error propagation.

The ΔG was calculated by integrating the areas of lipid mixtures over pressures Π = 5, 10, 15, 20, and 25 mN/m according to Grzybek et al. (8). Error bars were produced based on error propagation.

Dpop model development

A CHARMM-compatible model for Dpop was developed using the automated atom typing and parameter assignment pipeline CGenFF (9). CHARMM topology and parameter files are provided as Data S1.

Simulation composition and construction

Simulation systems contained either DOPC or POPC (unsaturation at either the Δ9 or Δ11 position) and one of either Dpop or Chol. All initial configurations were built using the CHARMM-GUI webserver (10–12). Systems containing atypical unsaturated chains (i.e., Δ11) were generated by first building a binary mixture of the corresponding Δ9 lipids (DOPC or POPC) with either Dpop or Chol and then "mutating" the unsaturated chain(s) to move the double bond to the appropriate position.

All simulations contained approximately 550 lipids per leaflet and at least 50 TIP3P (13) water molecules per lipid. All lipids were modeled with the CHARMM36 force field (12) except Dpop, which was modeled using the CHARMM general force field, with atom types determined by the paramchem server (Gromacs topology file is provided in the Data S1). Initial dimensions in the membrane plane were about 17.5 × 17.5 nm, containing approximately 270,000 atoms.

Four different binary mixtures were simulated: Δ9-DOPC:Dpop, Δ11-DOPC:Dpop, Δ9-POPC:Dpop, and Δ11 POPC:Dpop. Each binary mixture was simulated at four different compositions: 95:5, 85:15, 70:30, and 50:50. Each binary system was simulated for 500 ns of production simulation as described below. Four additional controls were simulated without any sterol or hopanoid, each for 50 ns of production simulation as described below: Δ9-DOPC, Δ11-DOPC, Δ9-POPC, and Δ11-POPC. The final simulation snapshots for the 50:50 Dpop with either Δ9-DOPC or Δ11-DOPC are shown in Fig. S1.

Equilibration and production simulations

Each system was prepared individually for production simulation through a series of minimization and heating steps as provided by the CHARMM-GUI equilibration protocol: 1) steepest descent to minimize the initial configuration; 2) 125,000 steps of leapfrog dynamics with a 1 fsec timestep and velocities reassigned every 500 steps; 3) 125,000 steps of leapfrog dynamics with a 1 fsec timestep, pressure controlled by the Parinello-Rahman barostat (14) and velocities reassigned every 500 steps, and then 4) a total of 750,000 steps of leapfrog dynamics with a 2 fsec timestep and hydrogen positions constrained by LINCS (15), pressure controlled by the Parinello-Rahman barostat (14), and velocities reassigned every 500 steps. During equilibration, double bonds were restrained in the *cis* configuration to prevent isomerization; these restraints are gradually reduced during the final three stages of the equilibration protocol. Production simulations (NPT ensemble) were integrated with leapfrog using the Parinello-Rahman (14) barostat to control pressure (time constant 5 ps; compressibility 4.5e−5 bar−1; coupled semi-isotropically to allow independent fluctuation of the in-plane and normal directions) and temperature controlled using Nose-Hoover (16,17) (time constant 1 ps) at a temperature of 298 K. Hydrogens were constrained with LINCS (expansion order 4), a 2 fsec timestep was used, short range electrostatics were computed directly within 1.2 nm, and long-range electrostatics were computed every timestep using particle mesh Ewald (18,19) with a grid spacing of 1 Å and cubic interpolation. Long-range dispersion was smoothly truncated over 10–12 nm using a force-switch cutoff scheme. Simulations were performed with Gromacs 2020.4.

Calculation of simulation observables

The distribution of angles between either Dpop or Chol and the membrane normal was computed, defining the orientation of both by a vector from atom C24 to atom O3. The locations of methyl groups in Dpop or Chol

along the direction normal to the membrane were recorded and compiled into histograms with a bin size of 0.87 Å. Deuterium order parameters were obtained from the simulations via

$$S_{CD} = \frac{1}{2}\langle 3\cos^2(\phi) - 1\rangle, \qquad \text{(Equation 2)}$$

where ϕ is the angle between the C–H bond vectors and the membrane normal at each position along the hydrocarbon chain. To obtain a single number that reports ordering for each chain, the S_{CD}s were averaged over neighboring carbons with the highest S_{CD} values, i.e., the order parameter "plateaus." These were defined as carbon numbers 4–8 so that the same carbon positions are averaged for each hydrocarbon chain. The location of each lipid is defined by the center of geometry of the C2, C21, and C31 atoms, and the location of the Chol/Dpop is defined by the O3 atom, and then a Voronoi construction is built around these points in the plane parallel to the membrane surface.

Cell culture

M. florum L1 strains were grown in a modified, lipid-free SP4 media with components as follows (per 1 L): 10 g Bacto Tryptone, 5.3 g Bacto Peptone, 3.5 g PPLO, 5.95 g BSA, 2 g Yeastoleate, 5 g D-glucose, 3.15 g sodium bicarbonate, 0.05 g L-glutamine, 0.645 g penicillin G-sodium salt, and 11 mg phenol red (pH 7.0). A lipid diet was added separately prior to passaging at the following concentrations: 5 mg/L Dpop and Chol, 25 mg/L egg sphingomyelin, 10 mg/L palmitic acid, and 12.5 mg/L Δ9- and Δ11-PC for the corresponding diets. Cells were grown in glass flasks and incubated at 30°C with shaking at 60 rpm. Growth was recorded using phenol red media pH detection through absorbance at 562 nm using a 10 mm cuvette (DeNovix DS-11 FX+). The growth rate was defined as the negative of the slope of the linearly fitted trendline in the indicative range of phenol red (OD_{562nm} from 0.75 to 0.4).

Membrane incorporation

Cells were collected in early exponential stage and centrifuged (5000 rcf, 7 min, 30°C). Supernatant was discarded, and the cell pellet was washed with wash buffer (200 mM NaCl, 25 mM HEPES, 1% glucose [pH 7.0]) and centrifuged (5000 rcf, 7 min, 30°C). The collected pellet was then subjected to a Bligh Dyer extraction (20). Briefly, the pellet was homogenized in a mixture of water:chloroform:methanol in 0.8:1:2 ratio and sonicated for 2 min. Subsequently, water and chloroform were added in 1:1 ratio. The mixture was centrifuged at 2000 rcf for 30 s in a mini centrifuge to promote phase separation. The lower, organic fraction containing lipids was collected and transferred to a fresh tube. The total lipid extract was then deposited on a silica gel plate (Supelco) and placed in a glass chamber. Chromatography was performed using chloroform as the running phase. After the run, the plate was dried and stained using 8% copper sulfate in 3% phosphoric acid solution and heated until visible bands were observed. Images were captured using a GelDoc (Biozym Azure c600) and analyzed using ImageJ.

Membrane osmotic shock

Cells were collected in the early exponential stage and centrifuged (5000 rcf, 7 min, 30°C). The collected pellet was resuspended in a serial dilution of 0%, 20%, 40%, 60%, 80%, and 100% of wash buffer (200 mM NaCl, 25 mM HEPES, 1% glucose [pH 7.0]). The suspension was stained with 10 μM propidium iodide and added to a 96-well plate. Fluorescence emission was recorded using a Tecan Spark fluorescence reader, with excitation at 529–549 nm and emission at 609–629 nm. The fraction of cell lysed was calculated by normalizing the signal of each sample to the 0% and 100% wash buffer sample. Figs. 3 *c* and 3 *e* represents the fraction of cells lysed in 80% of the wash buffer.

RESULTS

Since unsaturation changes the interaction between Chol/Dpop and phospholipids, we hypothesize that moving the position of double bonds along the phospholipid acyl chain can help us probe Chol/Dpop ordering, thus revealing key structural features needed for ordering. We also aim to draw a comparison between the ordering of Chol and Dpop to showcase a constraint in the evolution of sterol- and hopanoid-containing lipidomes.

We first determined the ordering of phospholipid chains by measuring monolayer surface pressure vs. area isotherms. Similar isotherms were obtained for PC phospholipids with a double bond at one of three positions, Δ6, Δ9, or Δ11 (chemical structures are shown in Fig. 1 *a*), suggesting similar packing in the pure membranes regardless of the isomer (Fig. 1 *b*). The differences in the molecular areas of the three isotherms were less than 3%. At 30 mN/m, the MMAs of Δ6-, Δ9-, and Δ11-PC were 65.3, 66.0, and 64.3 $Å^2$/molecule, respectively. This suggested that Δ9-PC was the largest, followed by Δ6-PC, and Δ11-PC was the smallest, in accordance with a previously reported molecular dynamics simulation (21,22). The isotherms differ, however, for binary mixtures of either Chol or Dpop in a manner dependent on both terpenoid choice and double bond position. To better quantify this difference, we calculated the condensing effect of the terpenoid on each lipid, as well as the free energy of mixing (ΔG_{mix}) the phospholipid and terpenoid (see the materials and methods for definitions and details). A positive condensing effect is obtained when the terpenoid orders lipid chains and increases lipid packing, which Chol consistently exhibited regardless of the double bond position. In contrast, Dpop only condenses the Δ11 isomer, does not noticeably affect the Δ6 isomer, and has a negative condensing effect on the Δ9 isomer. These observations are mirrored by ΔG_{mix}, which reflects the thermodynamic balance between lipid-lipid interactions and mixing entropy. Chol has favorable interaction with all three lipid isomers ($\Delta G_{mix} < 0$), which progressively increases as the double bond moves away from the headgroup. On the other hand, Dpop's interaction with PC varies based on the double bond positions. While Dpop was ideally mixed with Δ6-PC ($\Delta G_{mix} = 0$), the mixing of Dpop and Δ9-PC was unfavorable ($\Delta G_{mix} > 0$), and only with Δ11-PC was the $\Delta G_{mix} < 0$. The distinction between Dpop's interaction with Δ9-PC and Δ11-PC is intriguing, as the double bonds are just two carbons apart.

To gain molecular insight into the differential ordering of Δ9 and Δ11-PC by Dpop, we performed molecular dynamics simulations of bilayers of these binary mixtures (details are in the materials and methods). Fig. 2, *a–c*, reports the 2H NMR chain order parameters (S_{CD}) for several different binary mixtures of Dpop with phospholipids. S_{CD} is defined in Eq. 2 in the materials and methods; larger values indicate more ordered chains. A model for Dpop compatible with the CHARMM36 family of force fields was built as described in the materials and methods and first tested by simulating binary mixtures

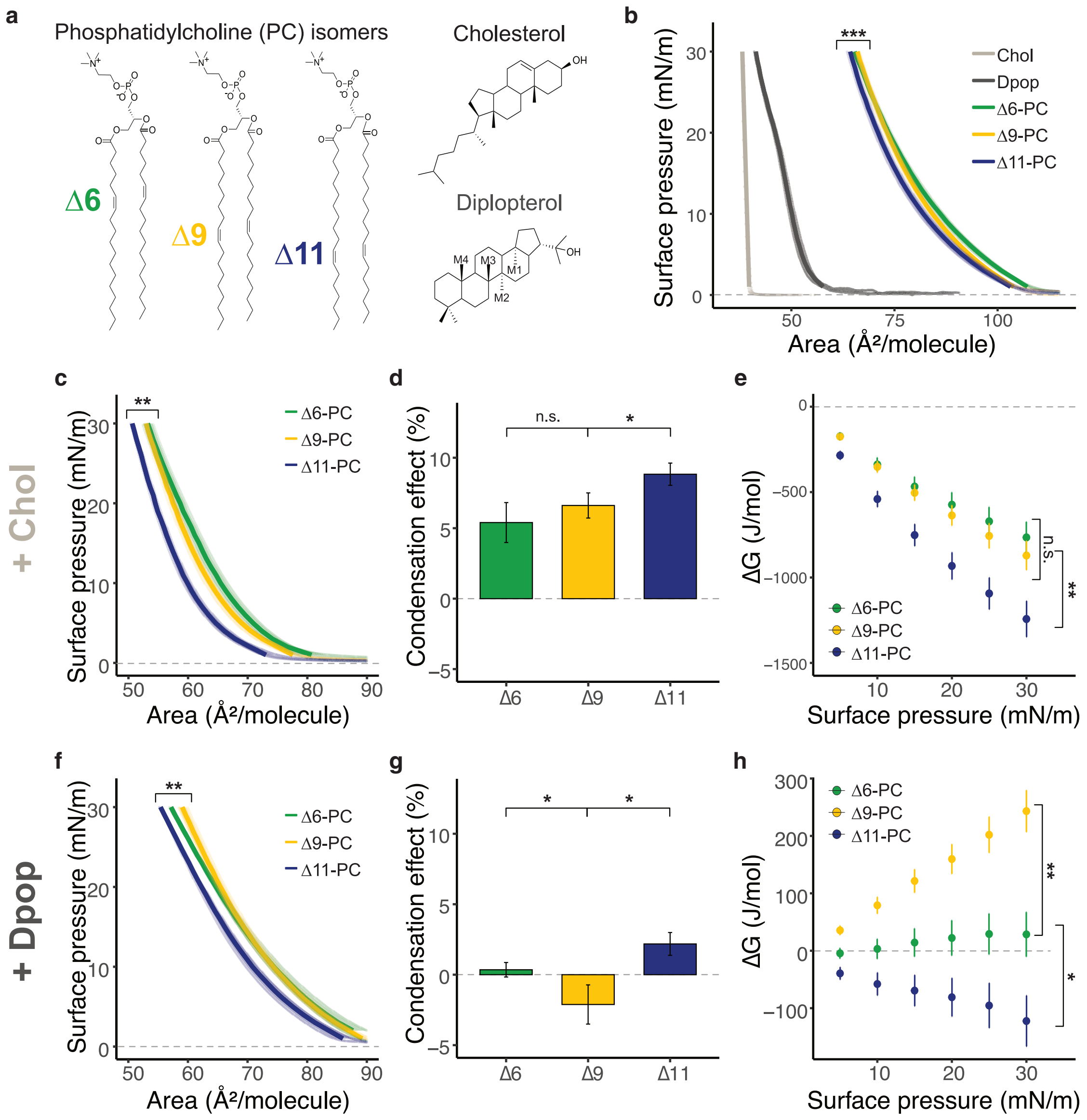

FIGURE 1 PC isomers interact differently with Chol and Dpop. While Chol ordering of PC increases as the double bond position is shifted further from the headgroup, Dpop only exhibits an ordering effect with Δ11-PC. (*a*) Chemical structure of di-unsaturated PC isomers, Chol, and Dpop and (*b*) isotherms of the corresponding lipids at 20°C, ***($F = 12.4$, $p < 0.0005$). (*c*) Isotherms of PC isomers mixed with Chol (2:1) at 20°C. **($F = 7.75$, $p < 0.005$) MANOVA. (*f*) Isotherms of PC mixture with Dpop (2:1) at 20°C. **($F = 8.18$, $p < 0.005$) MANOVA. (*d* and *g*) Energy of interaction (ΔGmix) of lipid pairs with Chol and Dpop, respectively, during compression. Error bar represents standard deviation. n.s. $p > 0.5$ and *$p < 0.05$, unpaired *t*-test. (*e* and *h*) Condensing effect of Chol and Dpop on PC isomers calculated at 30 mN/m. Error bar represents standard deviation. n.s. $p > 0.5$, *$p < 0.05$, and **$p < 0.005$, unpaired *t*-test. To see this figure in color, go online.

with DPPC (Fig. 2 *a*), obtaining an ordering effect similar to that observed in experiments on giant unilamellar vesicles (7).

We next considered binary mixtures with lipids with saturated chains at the sn-1 position and monounsaturated chains (Δ19 or Δ11 isomers) at the sn-2 position (Fig. 2 *b*). As expected, the saturated chains were more ordered by Dpop than the unsaturated chains and were ordered similarly regardless of which isomer was present in the other chain. Comparing the unsaturated chains, the Δ11 isomer was significantly more ordered than the Δ9 isomer. This trend was reproduced in PC with unsaturation in both acyl chains (Fig. 2 *c*), where we again observed that the Δ11 isomer was significantly more ordered. In summary, the position of the double bond was critical in determining the acyl chain's order when interacting with Dpop, mirroring the observations in the monolayer experiments and supporting a model in which the unsaturation position has a significant effect on condensation in fluid lipid bilayers.

In prior work, Martinez-Seara et al. found that the positioning of the double bond relative to the methyls protruding

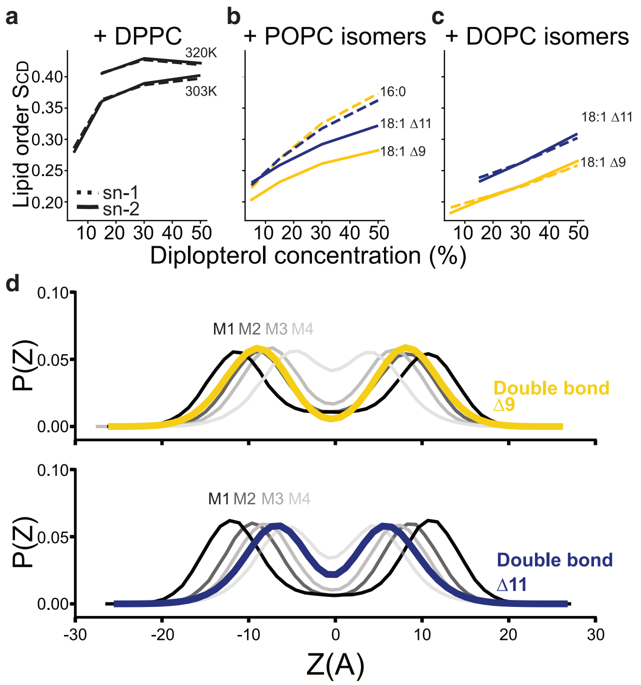

FIGURE 2 Molecular dynamic simulations show Dpop orders 18:1 Δ11 more efficiently than 18:1 Δ9, with a reduced ordering effect corresponding to the overlapping distribution of Dpop's methyl groups and PC's double bond. (*a*) Lipid order parameter of the saturated DPPC as a function Dpop concentration in its gel state (at 303 K) and liquid state (at 320 K). (*b*) Lipid order parameter of monounsaturated POPC isomers as a function Dpop concentration, where lipid order increases most significantly for saturated chains, then 18:1 Δ11, and the least for 18:1 Δ9. (*c*) Lipid order parameter of Δ9-PC and Δ11-PC as a function Dpop concentration, where Dpop orders Δ11 chain more effectively than Δ9. (*d*) Likely distribution P(Z) of PC isomer's double bond and Dpop's methyl groups over the membrane's *z* axis (perpendicular to the membrane surface). The double bond in Δ9-PC overlaps with Dpop M2, preventing efficient lipid packing, while no Dpop methyl group overlaps with the double bond of Δ11-PC. To see this figure in color, go online.

from Chol controls its condensing effect (23). We therefore performed a similar analysis, comparing the location of the PC lipid's double bond to the positions of methyl groups extending from the Dpop ring structure (annotated in Fig. 1 *a*) in our simulations. Fig. 2 *d* reports the distribution of double bonds of the hydrocarbon chains and Dpop methyl groups along the membrane normal, with $z = 0$ being the center of the bilayer. The distribution of Δ9-PC's double bond overlaps almost exactly with that of methyl group M2, while the Δ11-PC double bond falls in between the M3 and M4 methyl groups. The alignment between the π bond and the methyl group might explain the reduced condensing effect observed for Δ9-PC, while Dpop and Δ11-PC interacted more favorably (23). This interplay between lipid structure and membrane biophysics provides insights that could be extended to other sterols and hopanoids, possibly laying a path for predicting how hopanoid/sterol and phospholipid structure collectively influence membrane properties.

To investigate how this variation in lipid-lipid interaction might influence biomembrane function, we employed *M. florum* as a living model system. *M. florum* is a mollicute with no cell wall and a minimal genome (24). With limited machinery, *Mesoplasma* cannot synthesize its own lipids and relies on supplemented lipids from the media, offering a straightforward way to manipulate its lipidomes. By introducing either Δ9- or Δ11-PC to its lipid diet, we can create two identical biological membrane systems differing only in their unsaturation site. We then investigated this system to explore the effect of lipid-lipid interactions on a cellular scale.

Traditionally cultured with an undefined lipid diet in serums, we first test *Mesoplasma*'s ability to grow in a defined lipid diet. Like other Mollicutes, *M. florum* can take up lipids provided in the media. However, they are not capable of synthesizing or remodeling fatty acids, nor can they remodel the double bond position of phospholipids (25). Fig. 3, *a* and *b*, reports the growth rate and cellular lipid content, respectively, suggesting the *Mesoplasma* can both grow on and incorporate Chol and Dpop into their membrane. Since sterol ordering has previously been associated with membrane robustness (2), we tested *Mesoplasma* membrane robustness with hypoosmotic shock. Live cells are subjected to hypoosmotic conditions, forcing cells to rapidly expand. As the membrane is stressed and ruptures, exposed cellular DNA is stained by propidium iodide. By quantifying the fluorescence intensity, we estimated the fraction of cells lysed due to osmotic shock, inferring membrane robustness. Fig. 3 *c* shows the cell's susceptibility to osmotic shock when supplied with different lipid diets. When cells were supplied with Chol, the addition of Δ9- or Δ11-PC did not produce significant changes in cellular robustness. However, cells fed with Δ9-PC and Dpop exhibited higher susceptibility to lysis than cells fed with Δ11-PC and Dpop. These data suggested that the unfavorable interaction between Dpop and Δ9-PC counteracts Dpop's ability to bolster membrane robustness.

DISCUSSION

Chol and Dpop are both prevalent lipids, accounting for a substantial part of their respective membrane composition (26,27). Both of these tri-terpenoids condense monounsaturated lipid membranes but do so in a manner that depends on the location of the double bond position. The condensing effect of Chol is well established and has been shown to be isomer dependent in prior simulation work from Martinez-Seara et al. (23). The data presented above show that Dpop has the strongest condensing effect on Δ11-PC but is not as effective at condensing chains as Chol. Simulations indicate that Dpop's interaction with unsaturated lipids is significantly hindered by having multiple methyl groups extending from both sides of the cyclic ring structure. Notably, when the double bond resides at the Δ9 position and

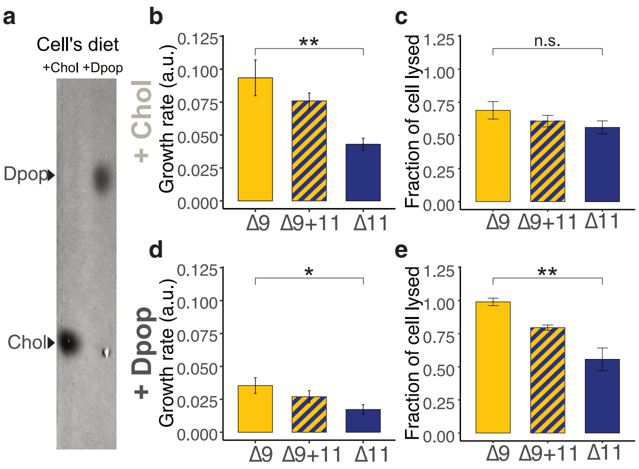

FIGURE 3 Dpop and Δ11-PC enhance robustness of *Mesoplasma florum* to hypoosmotic shock compared with Dpop and Δ9-PC. (*a*) Chol and Dpop were incorporated into *Mesoplasma* membranes according to their respective diets. (*b* and *d*) Growth rate of *M. florum* on different diets with Chol and Dpop, respectively. **(F = 24.4, $p < 0.005$). *(F = 10.6, $p < 0.05$). Analyses were performed using one-sided ANOVA with Tukey's post hoc test. (*c* and *e*) Membrane robustness reflected by the fraction of cell lysed when subjected to hypoosmotic shock on a diet with Chol and Dpop, respectively. n.s.(F = 1.47, $p < 0.5$). **(F = 16.6, $p < 0.005$). Analyses were performed using one-sided ANOVA with Tukey's post hoc test. To see this figure in color, go online.

overlaps with Dpop's methyl group M2, it prevents effective Dpop-induced lipid packing. While the negative correlation between methyl group-double bond proximity and lipid ordering was also observed for Chol by Martinez-Seara et al. (23), in the case of Dpop, the number of methyl groups (six methyl groups, with four in the region of interest) is higher than Chol (two methyl groups), suggesting more possible instances for double bond-methyl groups coming into close proximity. Another factor worth noting is that all of the methyl groups on Chol reside on one side of the planar ring, leaving a flat "alpha face." This feature is characteristic of lipid-ordering sterols such as Chol and contributes to the more effective lipid ordering of Chol over its sterol precursors. In combination with the earlier simulation results for Chol from Martinez-Seara et al. (23), our observations highlight the power of using simulations to explore lipid-lipid interactions, especially in the case of less commercially available lipids like hopanoids. With careful consideration in model development, one can explore the chemical landscape of lipids and the consequences of their collective interactions.

From these observations, we hypothesized that the differentiation between Δ9 and Δ11 should be the most significant in hopanoid-bearing membranes. In *M. florum*, the favorable interaction of Δ11-PC and Dpop enhanced membrane resilience to osmotic shock compared to Δ9-PC. This result highlights how subtle changes to lipid structure can have striking consequences for cellular robustness and suggests a potential mechanism for osmoadaptation, for which hopanoids have been shown to play a critical role in soil and plant-associated bacteria (28,29). Indeed, multiple hopanoid-bearing bacteria have Δ11 as the monounsaturation site (30) instead of Δ9, which is in eukaryotes. The hopanoid-producing yeast *Schizosaccharomyces japonicus* also possess a Δ12-desaturase (31,32). Interestingly, in 2020, Chwastek et al. investigated the lipidome of a hopanoid-bearing organism *Methylobacterium extorquens* and found that the main unsaturation position was Δ11 instead of Δ9, with an additional Δ5 unsaturation upregulated in cold-adapted lipidome (27). Therefore, the double bond position could represent a modifiable lipidomic feature that cells can employ to homeostatically fine-tune the ordering effects of hopanoids.

The tolerance of Chol for varying double bond positions might be credited to its lack of methyl groups, with only two protruding from one side of the cyclic plane. Chol with its flat alpha face is the result of several sequential biosynthetic steps involving the removal and repositioning of methyl groups (2,33). Consequently, the first sterol product in the Chol biosynthesis pathway, lanosterol, orders saturated lipids and not the unsaturated POPC, similar to Dpop (34–36). This suggests that the evolution of the pathways for methyl group rearrangement in sterol-bearing organisms provided more flexibility to produce lipids with double bond positions optimized for orthogonal lipid-lipid or lipid-protein interactions. To fully explore this possibility, the lipid-ordering capacity of a broader range of hopanoid and sterol structures should be systematically studied. However, our results raise an intriguing possibility that the transition from hopanoid to sterol-containing lipidomes could have widened the chemical landscape available for cells to explore for tuning membrane properties.

SUPPORTING MATERIAL

Supporting material can be found online at https://doi.org/10.1016/j.bpj.2024.06.002.

AUTHOR CONTRIBUTIONS

H.N.A.N. designed and performed the experiments, wrote the draft, and edited the manuscript. L.S. performed the simulations and analysis and wrote the corresponding method section. E.L. designed the simulations and edited the manuscript. J.P.S. conceptualized the project, designed the experiment, and edited the manuscript.

ACKNOWLEDGMENTS

The authors thank Tomasz Czerniak, Lisa Junghans, Nataliya Safronova, Isaac Justice, Fabian Rost, and Helena Jambor for helpful discussions and feedback. This work was supported by the B CUBE of the Technische Universität Dresden, a Maria Reich Fellowship (to H.N.A.N.), a Deutsche Forschungsgemeinschaft grant (DFG; to J.P.S., Project #527181518), a German Federal Ministry of Education and Research grant (Bundesministerium für Bildung und Forschung – BMBF; to J.P.S., Project No.

03Z22EN12), and a Volkswagen Foundation "Life?" grant (to J.P.S., Project No. 93090). E.L. and L.S. were supported by NIH award RO1GM120351. This work used Expanse2 at the San Diego Supercomputer Center through allocation BIO230093 from the Advanced Cyberinfrastructure Coordination Ecosystem: Services & Support (ACCESS) program, which is supported by National Science Foundation grants #2138259, #2138286, #2138307, #2137603, and #2138296.

DECLARATION OF INTERESTS

The authors declared no competing interests.

REFERENCES

1. Yamaguchi, K., and M. Kinoshita. 1993. Highly stable lipid membranes from archaebacterial extremophiles. *Prog. Polym. Sci.* 18:763–804.
2. Mouritsen, O. G., and M. J. Zuckermann. 2004. What's so special about cholesterol? *Lipids.* 39:1101–1113.
3. Belin, B. J., N. Busset, …, D. K. Newman. 2018. Hopanoid lipids: From membranes to plant-bacteria interactions. *Nat. Rev. Microbiol.* 16:304–315.
4. Brocks, J. J., G. D. Love, …, S. A. Bowden. 2005. Biomarker evidence for green and purple sulphur bacteria in a stratified Palaeoproterozoic sea. *Nature.* 437:866–870.
5. Racolta, S., P. B. Juhl, …, J. Pleiss. 2012. The triterpene cyclase protein family: A systematic analysis. *Proteins.* 80:2009–2019.
6. Sáenz, J. P., D. Grosser, …, K. Simons. 2015. Hopanoids as functional analogues of cholesterol in bacterial membranes. *Proc. Natl. Acad. Sci. USA.* 112:11971–11976.
7. Sáenz, J. P., E. Sezgin, …, K. Simons. 2012. Functional convergence of hopanoids and sterols in membrane ordering. *Proc. Natl. Acad. Sci. USA.* 109:14236–14240.
8. Grzybek, M., J. Kubiak, …, A. F. Sikorski. 2009. A raft-associated species of phosphatidylethanolamine interacts with cholesterol comparably to sphingomyelin. A Langmuir-Blodgett monolayer study. *PLoS One.* 4:e5053.
9. Vanommeslaeghe, K., E. Hatcher, …, A. D. Mackerell, Jr. 2010. CHARMM general force field: a force field for drug-like molecules compatible with the CHARMM all-atom additive biological force fields. *J. Comp. Chem.* 31:671–690.
10. Jo, S., T. Kim, …, W. Im. 2008. CHARMM-GUI: A web-based graphical user interface for CHARMM. *J. Comput. Chem.* 29:1859–1865.
11. Lee, J., X. Cheng, …, W. Im. 2016. CHARMM-GUI input generator for NAMD, GROMACS, AMBER, OpenMM, and CHARMM/OpenMM simulations using the CHARMM36 additive force field. *J. Chem. Theory Comput.* 12:405–413. https://doi.org/10.1021/acs.jctc.5b00935.
12. Wu, E. L., X. Cheng, …, W. Im. 2014. CHARMM-GUI Membrane Builder toward realistic biological membrane simulations. *J. Comput. Chem.* 35:1997–2004.
13. Price, D. J., and C. L. Brooks. 2004. A modified TIP3P water potential for simulation with Ewald summation. *J. Chem. Phys.* 121:10096–10103.
14. Parrinello, M., and A. Rahman. 1981. Polymorphic transitions in single crystals: A new molecular dynamics method. *J. Appl. Phys.* 52:7182–7190.
15. Hess, B., H. Bekker, …, J. Fraaije. 1997. LINCS: A Linear Constraint Solver for Molecular Simulations. *J. Comput. Chem.* 18:1463–1472. https://doi.org/10.1002/(SICI)1096-987X(199709)18:12%3C1463::AID-JCC4%3E3.0.CO;2-H.
16. Nosé, S. A. 1984. Molecular dynamics methods for simulations in the canonical ensemble. *Mol. Phys.* 52:255–268.
17. Hoover, W. G. 1985. Canonical dynamics: equilibrium phase-space distributions. *Phys. Rev. A.* 31:1695–1697. https://doi.org/10.1103/PhysRevA.31.1695.
18. Darden, T., D. York, and L. Pedersen. 1993. Particle mesh Ewald: An N·log(N) method for Ewald sums in large systems. *J. Chem. Phys.* 98:10089–10092.
19. Essmann, U., T. Darden, …, L. Pederson. 1995. A smooth particle mesh Ewald method. *J. Chem. Phys.* 103:8577–8593.
20. Bligh, E. G., and W. J. Dyer. 1959. A rapid method of total lipid extraction and purification. *Can J Biochem. Physiol.* 37:911–917. https://doi.org/10.1139/o59-099.
21. Wassall, S. R., M. A. McCabe, …, S. E. Feller. 2010. Solid-State 2H NMR and MD Simulations of Positional Isomers of a Monounsaturated Phospholipid Membrane: Structural Implications of Double Bond Location. *J. Phys. Chem. B.* 114:11474–11483.
22. Martinez-Seara, H., T. Róg, …, R. Reigada. 2007. Effect of double bond position on lipid bilayer properties: Insight through atomistic simulations. *J. Phys. Chem. B.* 111:11162–11168.
23. Martinez-Seara, H., T. Róg, …, R. Reigada. 2008. Interplay of unsaturated phospholipids and cholesterol in membranes: Effect of the double-bond position. *Biophys. J.* 95:3295–3305.
24. Baby, V., J. C. Lachance, …, S. Rodrigue. 2018. Inferring the Minimal Genome of Mesoplasma florum by comparative genomics and transposon mutagenesis. *mSystems.* 3:1–14.
25. Safronova, N., L. Junghans, and J. P. Saenz. 2023. From hot to cold: dissecting lipidome adaptation in the minimal organisms Mycoplasma mycoides and JCVI-Syn3B. Preprint at bioRxiv. https://doi.org/10.1101/2023.11.10.566608v1.
26. Lorent, J. H., K. R. Levental, …, I. Levental. 2020. Plasma membranes are asymmetric in lipid unsaturation, packing and protein shape. *Nat. Chem. Biol.* 16:644–652.
27. Chwastek, G., M. A. Surma, …, J. Sáenz. 2020. Principles of Membrane Adaptation Revealed through Environmentally Induced Bacterial Lipidome Remodeling. *Cell Rep.* 32, 108165.
28. Tookmanian, E. M., B. J. Belin, …, D. K. Newman. 2021. The role of hopanoids in fortifying rhizobia against a changing climate. *Environ. Microbiol.* 23:2906–2918.
29. Tookmanian, E., L. Junghans, …, D. K. Newman. 2022. Hopanoids Confer Robustness to Physicochemical Variability in the Niche of the Plant Symbiont Bradyrhizobium diazoefficiens. *J. Bacteriol.* 204, e0044221-21.
30. Fulco, A. J. 1983. Fatty acid metabolism in bacteria. *Prog. Lipid Res.* 22:133–160.
31. Makarova, M., M. Peter, …, S. Oliferenko. 2020. Delineating the Rules for Structural Adaptation of Membrane-Associated Proteins to Evolutionary Changes in Membrane Lipidome. *Curr. Biol.* 30:367–380.e8.
32. Bouwknegt, J., S. J. Wiersma, …, J. T. Pronk. 2021. A squalene–hopene cyclase in Schizosaccharomyces japonicus represents a eukaryotic adaptation to sterol-limited anaerobic environments. *Proc. Natl. Acad. Sci. USA.* 118, e2105225118.
33. Bloch, K. E. 1983. Sterol, Structure and Membrane Function. *Crit. Rev. Biochem.* 14:47–92.
34. Urbina, J. A., S. Pekerar, …, E. Oldfield. 1995. Molecular order and dynamics of phosphatidylcholine bilayer membranes in the presence of cholesterol, ergosterol and lanosterol: a comparative study using 2H-13C- and 31P-NMR spectroscopy. *Biochim. Biophys. Acta.* 1238:163–176.
35. Smondyrev, A. M., and M. L. Berkowitz. 2001. Molecular Dynamics Simulation of the Structure of Dimyristoylphosphatidylcholine Bilayers with Cholesterol, Ergosterol, and Lanosterol. *Biophys. J.* 80:1649–1658.
36. Sabatini, K., J.-P. Mattila, and P. K. J. Kinnunen. 2008. Interfacial Behavior of Cholesterol, Ergosterol, and Lanosterol in Mixtures with DPPC and DMPC. *Biophys. J.* 95:2340–2355.

Biophysical Journal, Volume *123*

Supplemental information

Varying the position of phospholipid acyl chain unsaturation modulates hopanoid and sterol ordering

Ha Ngoc Anh Nguyen, Liam Sharp, Edward Lyman, and James P. Saenz

Supplemental Data

Fig S1. Final snapshots of snapshots from Dpop simulations. Left panel: 50:50 mixture of Dpop and Δ9-DOPC. Right panel: 50:50 mixture of Dpop and Δ11-DOPC. Phospholipids are rendered in gray with blue spheres marking the phosphates, and Dpop is rendered in cyan with red spheres marking the hydroxyl.

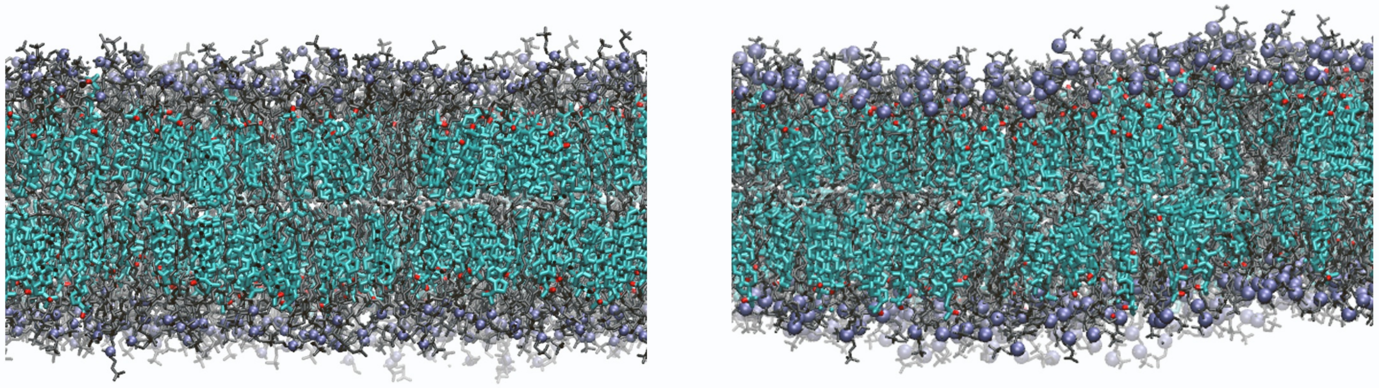

Fig S2. Raw data of 3 monolayers isotherm replicates.

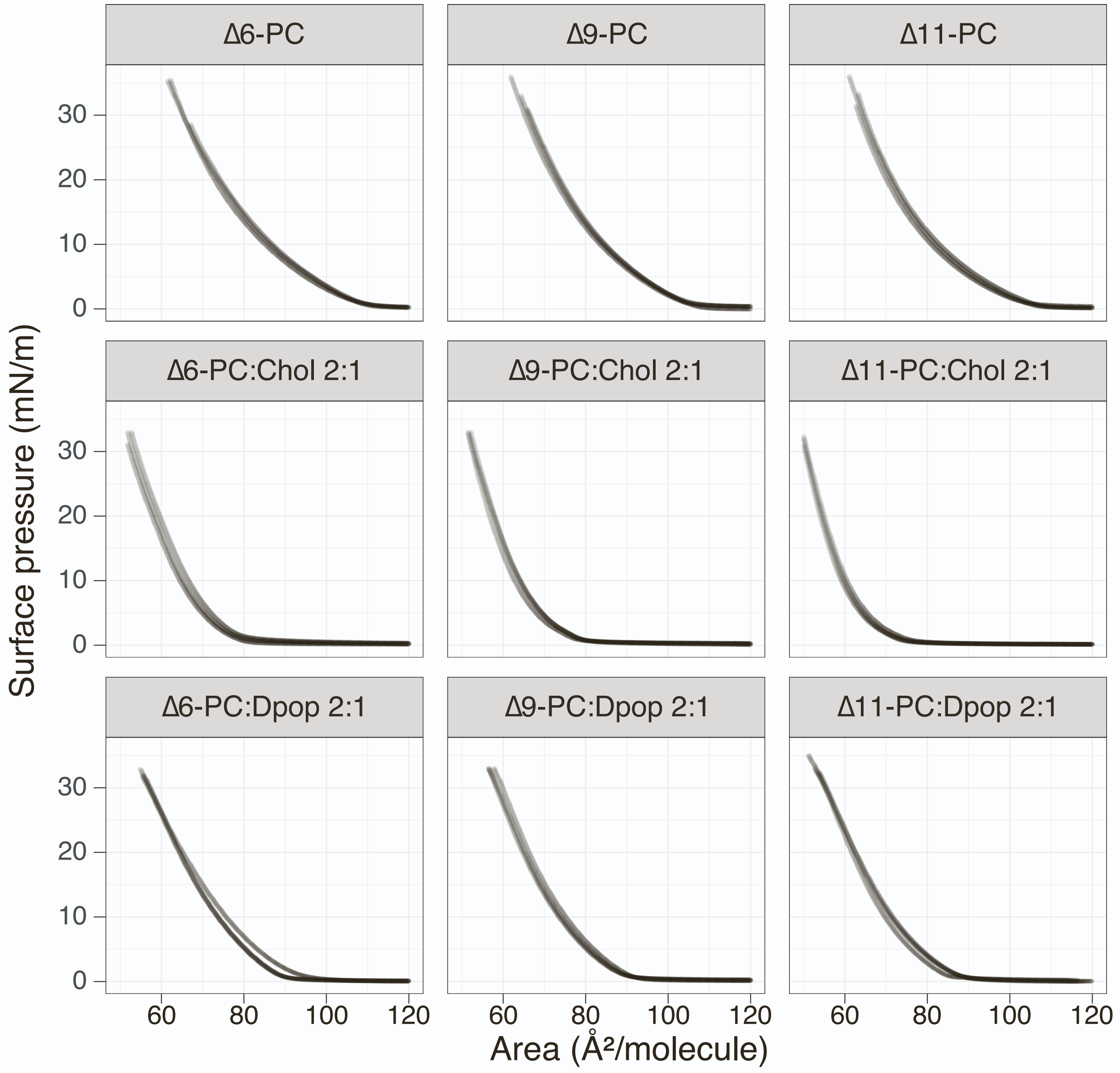

The isotherms were fitted to regression for statistical analysis with the following formula:

$$\text{Surface pressure} = \text{coef1} \times \frac{1}{\text{Area}^2} + \text{coef2}$$

Table S1. Coefficients calculated for each isotherm

Sample	Pure		1:2 Chol:PC		1:2 Dpop:PC	
	coef1	coef2	coef1	coef2	coef1	coef2
Δ6-PC	8.93E-05	4.87E-06	1.77E-04	6.25E-06	1.24E-04	5.91E-06
Δ6-PC	8.32E-05	5.07E-06	1.73E-04	6.02E-06	1.22E-04	6.10E-06
Δ6-PC	8.33E-05	4.89E-06	1.66E-04	5.85E-06	1.09E-04	6.49E-06
Δ9-PC	9.65E-05	4.64E-06	1.95E-04	5.67E-06	1.29E-04	5.47E-06
Δ9-PC	9.51E-05	4.50E-06	1.84E-04	5.84E-06	1.27E-04	5.43E-06
Δ9-PC	9.44E-05	4.43E-06	1.85E-04	5.58E-06	1.25E-04	5.16E-06
Δ11-PC	1.01E-04	4.75E-06	2.25E-04	5.75E-06	1.27E-04	6.62E-06
Δ11-PC	1.02E-04	4.99E-06	2.19E-04	5.77E-06	1.34E-04	6.24E-06
Δ11-PC	9.70E-05	4.77E-06	2.12E-04	5.88E-06	1.40E-04	6.37E-06

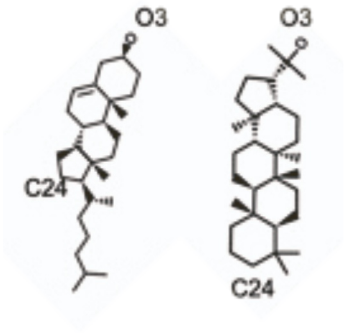

Fig S3. Structures of cholesterol (left) and diplopterol (right), with atoms used to define molecular orientation labeled.

Fig S4. Example growth curve through OD562nm read-out. The slope of the curve from 0.75 to 0.4 was used to calculate the growth rate (a.u.)

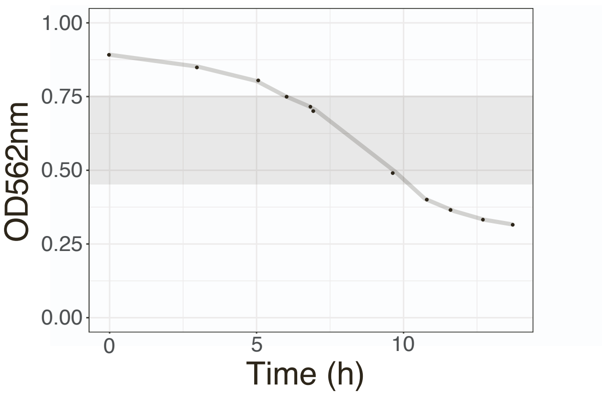

Supplement: Document S2. Article plus supporting material [file mmc3.pdf]
